# A rare case report of pituicytoma with biphasic pattern and admixed with scattered Herring bodies

**DOI:** 10.1186/s12957-020-01889-6

**Published:** 2020-05-27

**Authors:** Youde Cao, Yan Zeng, Xue Qin, Yiwen Tan, Min Zeng, Lijuan Wang, Xiaojing Cao, Lingfeng Zou, Chenglong Wang

**Affiliations:** 1 Department of Pathology, Chongqing Hospital of Traditional Chinese Medicine, 6 Seventh Panxi Branch Road, Jiangbei District, Chongqing, 400021 China; 2 Department of Pathology, College of Basic Medicine, Chongqing Medical University, 1 Yixueyuan Road, Yuzhong, Chongqing, 400016 China

**Keywords:** Pituicytoma, Perivascular pseudorosettes, TTF1, Pathology, Case report

## Abstract

**Background:**

Pituicytoma is a rare pituitary non-neuroendocrine tumour. The awareness of pituitary non-neuroendocrine tumours has gradually increased over the past several decades, but the knowledge of some histological variants of the tumours is limited, particularly in clinicopathological significance. Here, we report a rare case of pituicytoma variant.

**Case presentation:**

A 71-year-old man presented with sudden symptoms of stroke including urinary incontinence, weakness in right lower limb, and trouble speaking. Physical examinations showed a right facial paralysis. The radiological examinations eventually found a 1.7 × 1.4 × 1.3 cm sellar occupied lesion. After symptomatic treatment improved the symptoms, the patient underwent transsphenoidal resection of the pituitary mass. Histologically, the tumour contained hypocellular area and hypercellular area. The hypocellular area showed elongated spindle cells arranged in a fascicular pattern around small vessels and scattered Herring bodies; the hypercellular area showed a large number of pseudorosettes. Immunohistochemistrically, the tumour cells were positive for thyroid transcription factor-1, S100, and neuron-specific enolase. Neurofilament only showed a little positive in the hypocellular area, and silver impregnation was only noted in a perivascular distribution. The patient had no recurrence 4 months after the surgery.

**Conclusions:**

The rare variant of pituicytoma has a favourable prognosis. Moreover, it needs to be distinguished pituicytomas with pseudorosettes from ependymomas because of different prognosis. Lastly, Herring bodies may occasionally be seen in the pituicytoma, which could be a potential diagnostic pitfall.

## Background

The most common pituitary tumour is pituitary adenoma that is a neuroendocrine tumour, whereas pituitary non-neuroendocrine tumours are rare and include pituicytoma, spindle cell oncocytoma (SCO), granular cell tumour (GCT), and gangliocytoma according to the 2016 WHO classification of tumours of the central nervous system (CNS). The former three tumours have been found to arise from pituicytes and may constitute a spectrum of a single nosological entity because they are positive for thyroid transcription factor 1 (TTF1), a specific histogenetic marker of pituicytes [1]. The awareness of pituitary non-neuroendocrine tumours has gradually increased over the past several decades, but the knowledge of some histological variants of the tumours is limited, particularly in clinicopathological significance. To improve the awareness of the variants, we report a rare case of a pituicytoma with a biphasic pattern and admixed with scattered Herring bodies.

## Case presentation

**Fig. 1:**
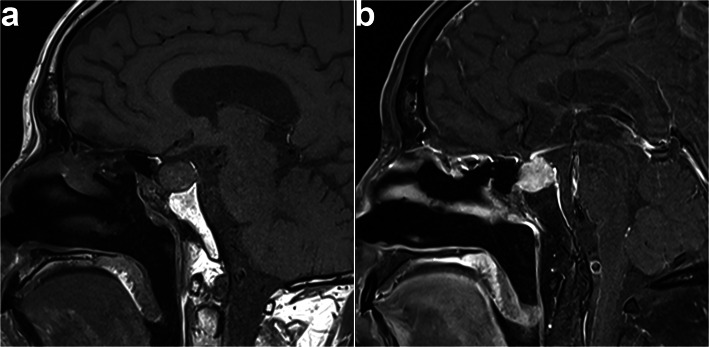
**a** T1-weighted MRI in the sagittal plane showed a space-occupying mass in the sellar region. **b** T1 postcontrast MRI in the sagittal plane showed heterogeneous enhancement in the mass

This patient was a 71-year-old man with a history of grade 2 hypertension for 30 years, and he presented with dizziness for a week. As sudden urinary incontinence, weakness in right lower limb, and trouble speaking, he underwent an emergency non-enhanced head computed tomography (CT) examination. The CT examination showed a slightly low-density area in the left frontal lobe and the left temporal lobe, which raised a suspicion for acute infarctions. The following contrast-enhanced head CT and magnetic resonance imaging (MRI) examinations showed no infarction, but a 1.7 × 1.4 × 1.3 cm sellar occupied lesion with heterogeneous enhancement (Fig. 1a, b). The patient had normal levels of pituitary hormones. Physical examinations showed the mouth drawn to the left side, the right nasolabial fold blunting, and the deviation of the protruded tongue toward the right side, which indicated a right facial paralysis. The symptoms were effectively relieved after the patient underwent the drug treatment including aspirin and atorvastatin for secondary prevention of stroke and ginkgo biloba extract for symptomatic treatment. The patient subsequently underwent transsphenoidal resection of pituitary mass on October 22, 2019.

**Fig. 2:**
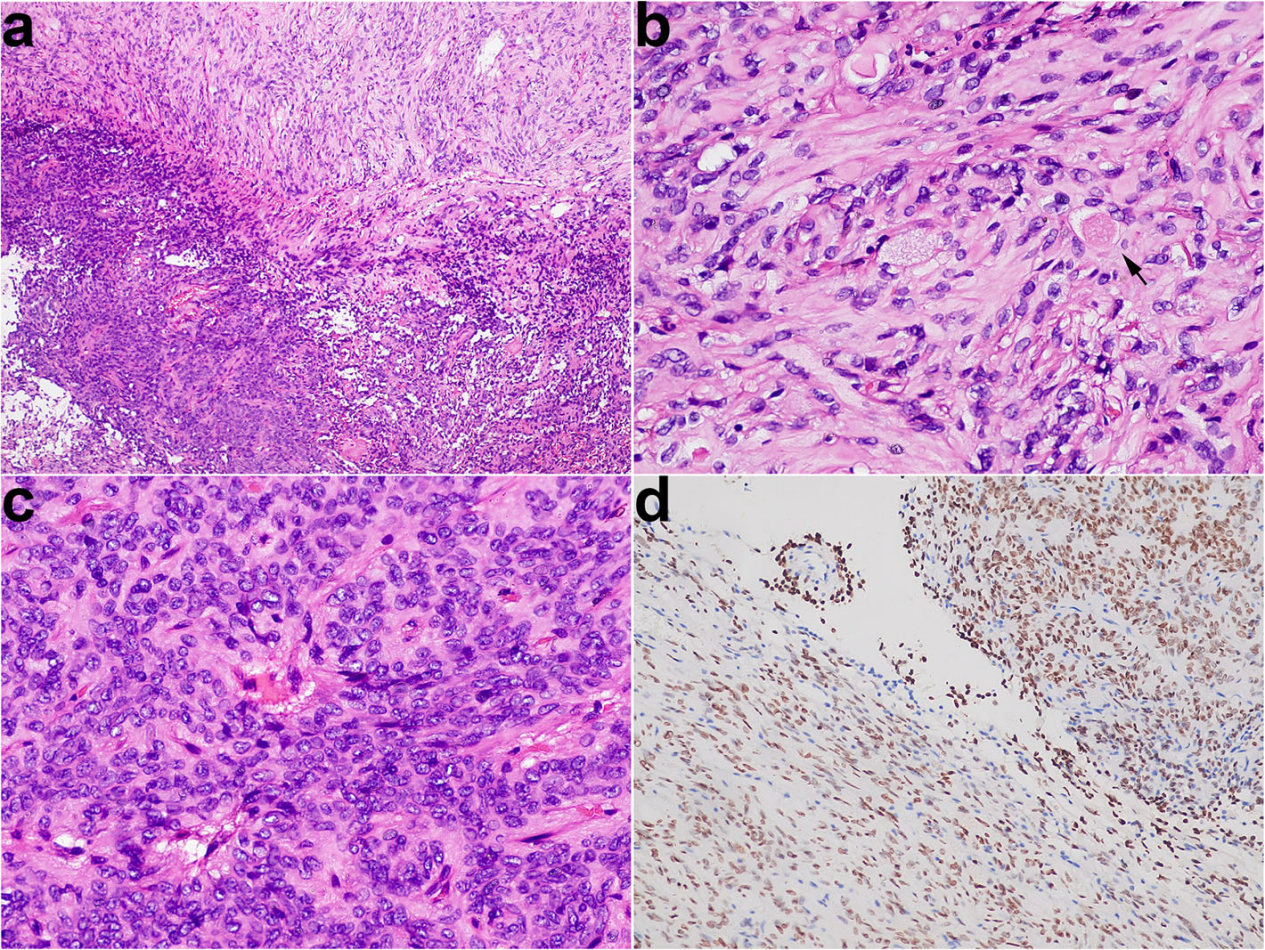
**a** The hypocellular area (top) and hypercellular area (bottom) showed apparent geographical separation. **b** In the hypocellular area, the spindle cells had blunted-ended to irregular nuclei with abundant, palely eosinophilic, fibrillary cytoplasm, and admixed with scattered Herring bodies (arrow). **c** In the hypercellular zone, a large number of pseudorosettes reminiscent of ependymoma. **d** Strong and diffuse nuclear staining for TTF1 in the hypocellular and hypercellular areas

**Fig. 3:**
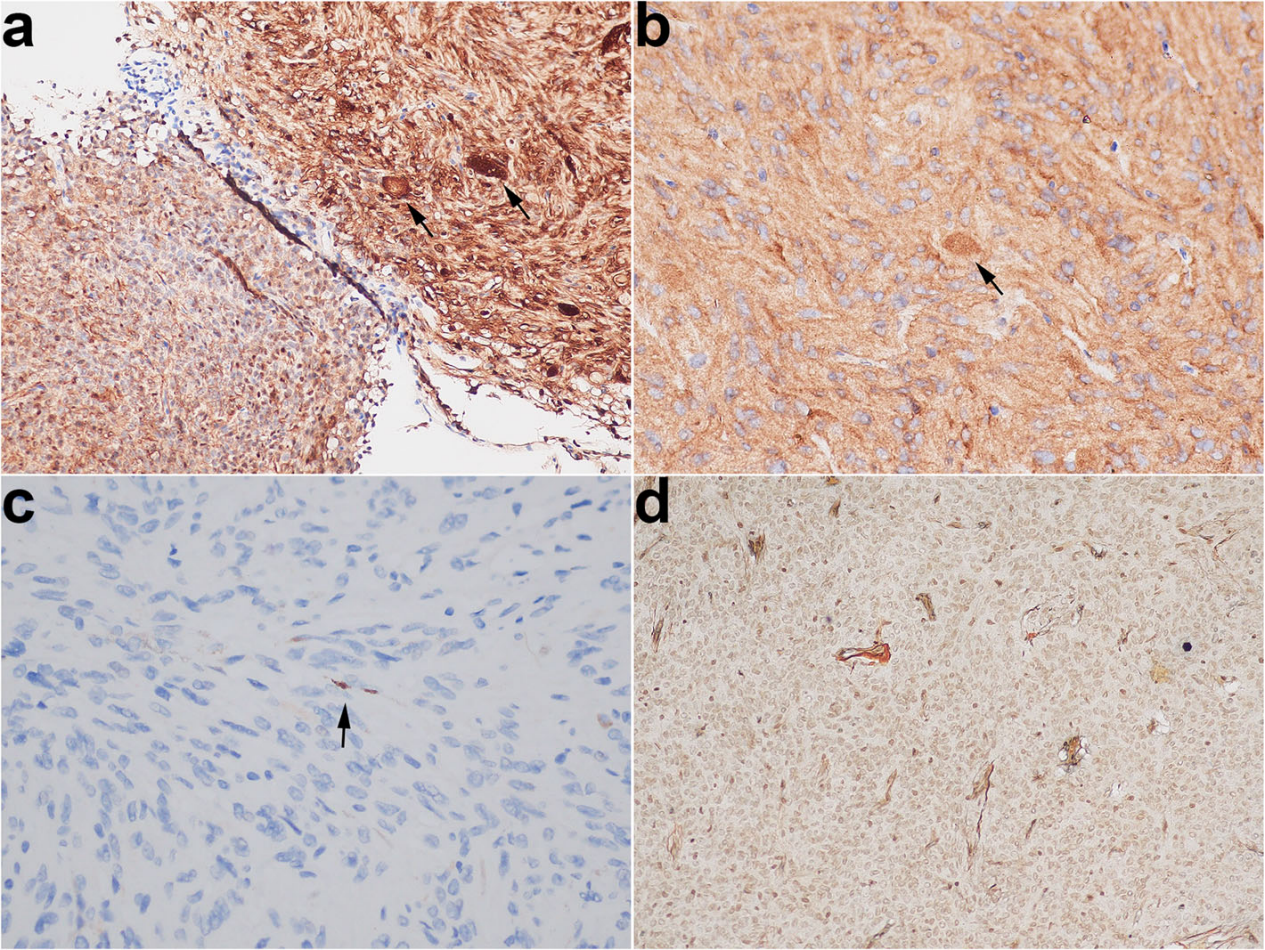
**a** S100 stains the nucleus and cytoplasm and highlights the Herring bodies (arrow). **b** NSE stains the cytoplasm and highlights the Herring bodies (arrow). **c** NF showed a little positive (arrow) in the hypocellular area. **d** Silver impregnation only noted in a perivascular distribution

The greyish-white colour resected specimen was about 1.5 cm in diameter. Histologically, this tumour contained hypocellular and hypercellular areas that showed apparent geographical separation (Fig. 2a). The hypocellular area showed elongated spindle cells arranged in a fascicular pattern around small vessels and scattered Herring bodies. The spindle cell had blunted-ended to irregular nuclei with abundant, palely eosinophilic, fibrillary cytoplasm (Fig. 2b). The hypercellular area was characterised by pseudorosettes in which the tumour cells showed crowding, overlapping the nucleus with speckled nuclear chromatin (Fig. 2c). Mitoses were not seen in the tumour. Immunohistochemistrically, the tumour cells showed diffuse nuclear expression of TTF1 (Fig. 2d). S100 (Fig. 3a) and neuron-specific enolase (NSE) (Fig. 3b) expressed in the tumour cells and Herring bodies. Neurofilament (NF) was completely negative in the hypercellular area but had a little positive in the hypocellular area (Fig. 3c). Silver impregnation was only noted in a perivascular distribution (Fig. 3d). Ki-67 showed extremely low proliferative index in the tumour. The other markers were negative, including glial fibrillary acidic protein, Olig2, SOX10, CD68, adrenocorticotropic hormone, thyroid stimulating hormone, growth hormone, prolactin, luteinizing hormone, follicle-stimulating hormone, SF1, PIT1, TPIT, cytokeratin, epithelial membrane antigen, CD68, and Galectin-3, neither was periodic acid–Schiff. The final diagnosis was pituicytoma with a biphasic pattern and admixed with scattered Herring bodies. The patient made a good postoperative recovery and had no recurrence at 4 months of MRI follow-up.

## Discussion and conclusions

Diligent clinical correlation, together with some appropriate immunohistochemical panel, is generally sufficient to reach the correct diagnosis of a rare tumour with an unusual morphology. Clinically, the symptoms of stroke pushed the radiologist to focus on looking for some suspicious cerebrovascular disease manifestation on the non-enhanced CT scan, resulting in missing the apparent lesion showed on the following contrast-enhanced CT and MRI examination: sellar tumour. Immunohistochemistrically, the tumour cells were positive for TTF1, S100, and NSE. According to the 2016 WHO classification of tumours of CNS, the tumour should fall within pituitary non-neuroendocrine TTF1-expressing tumours. Among the umbrella term encompassing pituicytoma, GCT, and SCO, pituicytoma was eventually picked out to diagnose the sellar tumour based on the following analyses.

GCT and SCO have been referred to constitute pituicytomas that are composed of tumour cells with lysosome-rich and mitochondrion-rich cytoplasm, respectively [1]. The lysosome is identified in practice by CD68 marker that belongs to the lysosome-associated membrane protein family of molecules [2] and consistently expresses in lysosome-rich cells [3–5]. Mitochondrion-rich cells usually show apparent eosinophilic cytoplasm, such as Hürthle cell [6], and gastric parietal cells [7]. Moreover, there is evidence that most of SCO are positive for epithelial membrane antigen and Galectin-3 [8]. In this case, the tumour cells are negative for the three markers and lack apparent eosinophilic cytoplasm, so the diagnosis of pituicytoma is appropriate. This pituicytoma presents some unusual morphological features that we will focus on in the following.

This pituicytoma rarely contains two different morphological areas. In the hypercellular area, a large number of pseudorosettes are reminiscent of ependymoma. The pattern presented in sellar tumours has been described in some literature, but the nomenclature is not well established owing to limited understanding of them, with different studies referring to the tumour as “ependymoma” or “pituicytoma with an ependymoma-like component” [9–13]. Compared with the pituicytoma that arose from pituicytes, which are associated with a favourable outcome according to the 2016 WHO classification of tumours of CNS, the ependymoma that arose from ependymal cells are defined as WHO grade II or III malignant tumours that have a variable clinical outcome, ranging from long-term disease-free survival after surgery to local recurrence to metastasis [14, 15]. Considering the different prognosis, ependymoma and pituicytoma should be distinguished. To distinguish the two that have different cells of origin, the best way is to perform a specific histogenetic immune marker of pituicytes: TTF1. As the marker is diffusely expressed in the tumour cells, the diagnosis of pituicytoma with an ependymoma-like component is confirmed in the hypercellular area. The hypocellular area is reminiscent of a normal neurohypophysis. In the neurohypophysis, silver impregnation or NF stain can demonstrate such axonal processes originated from the hypothalamus, traverse the pituitary stalk, to the perivascular zones of the posterior lobe [16]. However, the stains show almost complete loss of the axons in the hypocellular area. Moreover, the area shows that the cells have more abundant cytoplasm with mild nuclear atypia, and has a higher cellular density, than the neurohypophysis. The findings actually reflect the ability of tumour proliferation and invasion in the area, so the hypocellular area should be a part of the pituicytoma. This pituicytoma lacks a histological continuity between the two areas (as shown in Fig. 2a), so we speculate that it is multicentric.

Herring bodies, dilated terminal portions of neurosecretory axons from the hypothalamus and stored antidiuretic hormone and oxytocin, are regarded as a useful diagnostic clue of neurohypophysis according to the 2016 WHO classification. However, Herring bodies have been described in at least four cases of pituicytoma, including ours [12, 17, 18]. In this case, Herring bodies only presented in the hypocellular area. In the area, the tumour ability to destroy the axons is weaker than in the hypercellular area based on observing the NF stain. Therefore, we speculate that Herring bodies can be alive when the tumour invasive ability is rather weak. Moreover, the patient should have presented abnormal levels of antidiuretic hormone or oxytocin, or hormone-related symptom due to the abnormal Herring bodies lacked the connection of axons with the hypothalamus, but either this case or the other three cases have not. The underlying mechanism by which abnormal Herring bodies of pituicytoma never cause hormone imbalances warrants further study.

In conclusion, the rare variant of pituicytoma has a favourable prognosis. Moreover, it is necessary to distinguish the pituicytoma with pseudorosettes from the ependymoma because of different prognosis. Lastly, Herring bodies can occasionally be seen in a pituicytoma, which is a potential diagnostic pitfall.

## Data Availability

All the original data supporting our research are described in this article.

## Abbreviations

SCO: Spindle cell oncocytoma
GCT: Granular cell tumour
CNS: Central nervous system
TTF1: Thyroid transcription factor 1
CT: Computed tomography
MRI: Magnetic resonance imaging
NSE: Neuron-specific enolase
NF: Neurofilament
